# Lian Hua Qing Wen Capsules, a Potent Epithelial Protector in Acute Lung Injury Model, Block Proapoptotic Communication Between Macrophages, and Alveolar Epithelial Cells

**DOI:** 10.3389/fphar.2020.522729

**Published:** 2020-09-23

**Authors:** Qi Li, Qingsen Ran, Lidong Sun, Jie Yin, Ting Luo, Li Liu, Zheng Zhao, Qing Yang, Yujie Li, Ying Chen, Xiaogang Weng, Yajie Wang, Weiyan Cai, Xiaoxin Zhu

**Affiliations:** ^1^ Institute of Chinese Materia Medica, China Academy of Chinese Medical Sciences, Beijing, China; ^2^ School of Chinese Materia Medica, Capital Medical University, Beijing, China

**Keywords:** Lian Hua Qing Wen capsule, acute lung injury, macrophages and epithelial cells, endoplasmic reticulum stress, tumor necrosis factor-related apoptosis-inducing ligand

## Abstract

Besides pathogen evading, Acute Lung Injury (ALI), featuring the systematic inflammation and severe epithelial damages, is widely believed to be the central non-infectious factor controlling the progression of infectious diseases. ALI is partly caused by host immune responses. Under the inspiration of unsuccessful treatment in COVID-19, recent insights into pathogen–host interactions are leading to identification and development of a wide range of host-directed therapies with different mechanisms of action. The interaction unit consisting of macrophages and the alveolar epithelial cells has recently revealed as the therapeutic basis targeting ALI. Lian Hua Qing Wen capsule is the most effective and commonly-used clinical formula in treating respiratory infection for thousands of years in China. However, little is known about its relevance with ALI, especially its protective role against ALI-induced alveolar tissue damages. Aiming to evaluate its contribution in antibiotics-integrating therapies, this study pharmacologically verified whether LHQW could alleviate lipopolysaccharide (LPS)-induced ALI and explore its potential mechanisms in maintaining the physiology of macrophage-epithelial unit. In ALI mouse model, the pathological parameters, including the anal temperature, inflammation condition, lung edema, histopathological structures, have all been systematically analyzed. Results consistently supported the effectiveness of the combined strategy for LHQW and low-dose antibiotics. Furthermore, we established the macrophages-alveolar epithelial cells co-culture model and firstly proved that LHQW inhibited LPS-induced ER stress and TRAIL secretion in macrophages, thereby efficiently protected epithelial cells against TRAIL-induced apoptosis. Mechanistically, results showed that LHQW significantly deactivated NF-κB and reversed the SOCS3 expression in inflammatory macrophages. Furthermore, we proved that the therapeutic effects of LHQW were highly dependent on JNK-AP1 regulation. In conclusion, our data proved that LHQW is an epithelial protector in ALI, implying its promising potential in antibiotic alternative therapy.

## Introduction

Acute lung injury (ALI), characterized by increased alveolar epithelial and lung epithelial permeability, followed by alveolar flooding and systematic inflammation, was considered as a common pathological mechanism and happened in most infectious diseases (Quartin et al., 2009). ALI is often manifested as one of the most important mechanisms, which involves various tissues damage and eventually leads to multiple organ dysfunction (Oflazoglu et al., 2018). Notably, among all the pathological processes within the ALI network, recent studies have found that aberrant macrophage activation followed by disturbed phenotypic switch are all closely linked with pathological changes in the development of ALI. They promote the lung inflammation and tissue damage, as well as the damage of pulmonary vascular permeability, resulting in pulmonary edema, are all closely linked with pathological changes in the development of ALI (Gordon, 2005; Imai et al., 2008). Therefore, it is of great importance to confirm the relationship between ALI and tissue damage induced by macrophages, so as to provide new revelation in the treatment strategies of the respiratory infectious diseases system.

Disappointedly, in contrast to the essential roles of macrophages in ALI, drugs targeting macrophages regulation and tissue protection under inflammatory conditions are rarely mentioned at present. The treatment of pulmonary infectious diseases still focused on pathogen inhibition, frequently resulting in the upgrading of drug resistance and failure of treatment. This therapeutic dilemma has greatly inspired current researchers. Instead of simply pathogen eliminating, accumulating evidences have highlighted strategies focusing on immune-relevant mechanisms as represented by macrophage regulation and immunological balance remodeling of ALI. Particularly, coronavirus disease 2019, (COVID-19) is rampant spreading all over the world. Due to the poorly understanding about this novel virus and the lacking of specific anti-virus drugs it has become the biggest threat for clinicians in the world(Li and De Clercq, 2020). The lacking drugs of direct inhibition to the pathogen and non-pathogen treatment strategies have mostly contributed to an overwhelming threat of COVID-19. Hopefully, like a beam lightening the darkness, the traditional Chinese medicine (TCM), specialized in immune correcting and tissue protection under inflammatory conditions, plays decisive role in the pandemic controlling, which put another evidence for the importance and effectiveness of non-pathology-killing strategies against infectious respiratory diseases.

In ALI conditions, the pathogenesis of tissue damage, which can be seen as the indicator of pathological progress, is related to inflammatory reaction, cell apoptosis, necrosis and death. Particularly, tumor necrosis factor related apoptosis-inducing ligand (TRAIL) displays core role in regulating these processes and then contribute to pathological damage. Pathologically, the activation of proapoptotic and pro-necroptotic pathways, which can result in a structural disruption of the airway, is a major hallmark of pathological process controlled by TRAIL. In addition, TRAIL can also induce the disruption of the alveolar epithelial barrier by cell death, which significantly contributes to worsened unspecific tissue injury and disease severity in ALI(Tabas, 2010; Alessandri et al., 2013; Condamine et al., 2014; Huang et al., 2015). Recent reports further suggested that TRAIL expression is mediated by ERS, which is linked to CHOP, a popular marker for assessment of ERS. A number of investigators have reported that suppression of ER stress markedly reduced the LPS-induced lung injury. Mechanically, ERS-induced TRAIL expression is mediated by the JNK/AP-1 signaling pathway, nevertheless the production of TRAIL induced by ERS was negatively controlled by SOCS3 through inhibiting phosphorylation of c-Jun *via* the JNK pathway.

Lian Hua Qing Wen capsules (LHQW) is the most effective and commonly-used clinical formula in treating respiratory infection for thousands of years in China. It has approved by China Food and Drug Administration (CFDA). LHQW is composed of 11 herbs including Forsythia suspensa (Thunb.) Vahl (Lianqiao); Lonicera japonica Thunb. (Jinyinhua); Honey-fried Ephedra sinica Stapf (Mahuang); Prunus armeniaca L. (Kuxingren); Isatis tinctoria L. (Banlangen); Dryopteris crassirhizoma Nakai (Mianmaguanzhong); Houttuynia cordata Thunb. (Yuxingcao); Pogostemon cablin Benth. (Guanghuoxiang); Rheum palmatum L. (Dahuang), Rhodiola crenulata (Hook.f. & Thomson) H.Ohba (Hongjingtian); and Glycyrrhiza glabra L. (Gancao); along with menthol (Bohenao)and a traditional Chinese mineral medicine, Gypsum Fibrosum (Shigao). Chemically, a total of 61 compounds were unambiguously or tentatively identified and divided into flavonoids, phenylpropanoids, anthraquinones, triterpenoids, iridoids, and other types by ultrahigh-performance liquid chromatography coupled with diode array detection and quadrupole time-of-flight mass spectrometry analysis (Jia et al., 2015). Pharmacologically, the activities of individual components have been partially revealed (**Table 1**). Pharmacological studies of LHQW have shown that it has a clear inhibitory effect on the growth and spread of pulmonary infectious pathogens (Dong et al., 2014). Strikingly, LHQW capsule have shown therapeutic effects on inhibiting the COVID-19 based on its ability in significantly improving fever and cough symptoms in confirmed pneumonia patients. Recent clinical studies showed that disappearance rate of fever was 85.7% (57.1% in the control group), and the disappearance rate of cough was 46.7% (5.6% in the control group) after LHQW treatment.

**Table 1:** Effects of the components from Lian Hua Qing Wen capsule (LHQW) capsule.

Component	Effect	Reference
Flavonoids	Preventing Carcinogen Metabolic Activation, anti-oxidative Activity	(Li, 2003)
Phenylpropanoids	Anticancer, antiviral, anti-inflammatory, wound healing, and antibacterial	(Korkina et al., 2011)
Anthraquinones	Inhibit cellular proliferation, induce apoptosis, and prevent metastasis.	(Huang et al., 2007)
Triterpenoids	Defend against signal transducer, and activate transcription and angiogenesis	(Petronelli et al., 2009)
Iridoids	Anti-mutagenic, antispasmodic, anti-tumor, antiviral, immunomodulation	(Yamazaki et al., 1994)

The previous research of ALI were concentrated in direct killing for pathogens clearance. However, it lacked the evaluation and mechanism disclosure to the tissue damage in ALI. Based on the deficiencies, our studies showed a comprehensive understanding of LHQW. It is of great significance to expand the field and deepen the treatment thinking in treating respiratory infectious diseases. Our studies previously showed that LHQW can effectively ameliorate LPS-induced lung inflammation. In this current research, we focused on the core mechanism of pathological damages. We also evaluate the efficacy and potential molecular mechanism of LHQW in tissue protection and physiological reconstruction in the treatment of ALI. Importantly, the results of this study can provide some reference value for the treatment of ALI in humans.

## Materials and Methods

### Reagents

LianHuaQingWen capsules (LHQW) were from Shijiazhuang Yiling Pharmaceutical Co., Ltd. (Shijiazhuang, China, batch number: B1509001). DEX was from Li Sheng Pharmaceutical Inc. (TianJin, China). PS was obtained from Solarbio (Beijing, China), LPS (Escherichia coli 055: B5) and PMA were from Sigma (St. Louis, MO, USA). RPMI-1640 and Dulbecco’s minimum essential medium (DMEM) were from Gibco (Grand Island, NY, USA). Heat-inactivated fetal calf serum was obtained from HyClone (Logan, UT, USA). Methyl thiazolyl tetrazolium (MTT) and dimethyl sulfoxide (DMSO) were from Amresco (Solon, OH, USA). Annexin V-propidium iodide (AV-PI) was from BD Biosciences (Franklin Lakes, NJ, USA). Caspase3 and Bradford protein assay kits were from Beyotime (Shanghai, China). Primary antibodies against phospho-JNK (#4671), total-JNK (#9258), phospho-p65 (#3033), phospho-IKK (#2697), total-IKK (#2682), IKB-α (#11930), and GRP78 (#3177S) were from Cell Signaling (Danvers, MA, USA); CHOP (ab11419), DR5 (ab199357), and SOCS3 (ab16030) were from Abcam (Cambridge, UK); β-actin (66009-1) and GAPDH (60004-1) were from Proteintech (Rosemont, IL, USA); total-p65 (sc33020), ICAM1 (sc-8439), and VCAM1 (sc-1504) were from Santa Cruz Biotechnology (Dallas, TX, USA); and Na,K ATPase (-369) was from Millipore (Burlington, MA, USA). Enzyme-linked immunosorbent assay (ELISA) kits for TRAIL, tumor necrosis factor (TNF)-α, interleukin (IL)-1β, and IL-6 were purchased from DAKEWEI (Shenzhen, China). All other chemical reagents meet the reagent specification standards.

### Sample Preparation

The powder of LHQW capsules (0.4 g) was accurately weighed and extracted with 60% methanol-water (v/v) solution (20 ml) in an ultrasonic water bath for 30 min at room temperature. The supernatant solution was diluted with the same amount of water and then centrifuged for 10 min at 14,000 r/min. All the obtained solutions were filtered through 0.22 μm syringe filter before the UPLC analysis.

### Animal Model of ALI and Experimental Groups

A total of 100 ICR male mice (6–8 weeks old, 18–20 g weight) were purchased from Si Bei Fu (Beijing, China). All mice were kept in an environment of 24°C ± 1°C and 65% humidity, under a 12-h light cycle for 3 days before starting the experiments. Water and food were available ad libitum. All experiments were carried out according to the guidelines for proper conduct of animal experiments published by the Science Council of Beijing, as well as the ARRIVE (Animal Research: Reporting of *In Vivo* Experiments) guidelines for animal research.

For each experiment, age-and weight-matched groups of mice were used. After the last drug treatment, mice were anesthetized with 4% chloral hydrate (0.4 g.kg^-1^) for 20 min and placed in the supine position on the surgical board(the statement of the use of chloral hydrate was described as following: It is undeniable that the application of chloral hydrate possesses potential risks and serious adverse effects during operative anesthesia, which include, but not limited to, post-operative ileus.). The route of administration was intraperitoneal injection. Then the trachea was exposed by a cervical incision. The whole courses were supported by ventilator. Control mice were instilled with saline, and others were treated with LPS (5 mg·kg^-1^). All of the above processes were performed on a ventilator. The mice were treated daily for one week prior to the LPS administration. Additionally, the operations were carefully monitored and under strict experimental control. The anesthesia-only control group and sham-operative group were both set to avoid interference from chloral hydrate -induced unintended adverse effects. During and after the surgery, we also paid much attention to the living conditions of mice once the chloral hydrate was administrated and such post-operative care were maintained until the mice were recovered from anesthesia. For example, we put the mice on the heating pad, which set to keep the body temperature at 37°C. Besides, for the mice receiving operations, their cages were covered with soft cotton pads to prevent the unintended injury.

LHQW and dexamethasone (DEX) were suspended in 0.5% (w/v) carboxymethylcellulose solution and orally administered *via* gavage once daily (300, 600, and 1,200 mg.kg^-1^) from day 0 to day 7. Penicillin-streptomycin (PS) was injected intramuscular once daily (10, 2.5 ml.kg^-1^).

### Cell Culture and Cell Viability Assay

All cells were maintained in culture medium supplemented with 10% fetal bovine serum (Gibco) at 37°C in a humidified incubator (5% CO_2_) (SANYO, Osaka, Japan), with HPAepic and THP-1 cells in RPMI-1640 medium and RAW 264.7 cells in DMEM medium. MTT assay was used to detect living conditions of cells at 450 nm with a microplate reader. HPAepic were purchased in National Infrastructure of Cell line Resource. (According to the information provided by the company, HPAepic cells were not directly isolated from human tissues by us. Also, they were not the primary cells cultured by any companies. Instead, they were immortalized cell line and can be stably passaged *in vitro*).

### Detection of Wet/Dry Weight of Lung Tissues and Anal Temperature

The measurement was performed at room temperature of 24.0°C. The Anal temperatures of the mice were measured by using a digital, blunt-tipped stem thermometer(SH series), with Vaseline smearing on it, 2 h after surgery. According to the manufacturer`s instruction, half the length of the blunt-tipped stem was staying in the anus for 30 s and final temperatures were recorded when the temperature remains unchanged for 10 s. After the mice were euthanized, the lungs were removed and the wet weight was determined. The lung tissue was placed in an oven at 60°C for 48 h to obtain the dry weight. The ratio of the wet lung to the dry lung was calculated to assess tissue edema. (The arrive-guidelines of experimental-procedures were shown in **Table 2**).

**Table 2:** Arrive-guidelines of experimental-procedures.

Procedures	Euthanasia
Method of euthanasia	Anesthesia methods (The mice were maintained in a specific pathogen-free animal care holding room. It was evaluated monthly to monitor the colony for pathogen exposure. In addition, colony animals identified for euthanasia were monitored for pathogen exposure. Mice were housed in polycarbonate (19.56 cm × 30.91 cm × 14.92 cm) ventilated cages. Cages were changed twice weekly. The animal holding room was maintained under environmental conditions of 22 ± 1°C, relative humidity of 50% ± 10%, 20 air changes/h, and a 12:12-h light:dark cycle. Mice received pelleted rodent diet and water ad libitum.)
Pharmacological agent	Drug formulation was liquid, the dose was 1.2 g.kg-1, the concentration was 10% chloral hydrate (10g chloral hydrate in 100 ml 0.9% saline), the administration method was intraperitoneal injection
Any measures taken to reduce pain and distress before or during euthanasia	Every anesthetized mice was placed in dorsal recumbency on a specialized mouse board with heating elements. This board was placed on top of a 37°C heated recirculating water pad. rectal body temperature was monitored continuously.
Tissues collected post-euthanasia and timing of collection	Lung tissues were collected. Timing of collection was when the animal lost its righting reflex, stopped moving, or appeared to be unconscious.

### Alveolar Lavage and Alveolar Macrophage Preparation

Mice were firstly anesthetized with 4% chloral hydrate (0.4 g.kg-1) (intraperitoneal injection) for 20 min 24 h after surgery. After that, the trachea was fully exposed and can be clearly visualized. Then the lungs were lavaged three times with 1 ml saline each time. Alveolar lavage was centrifuged at 135 rcf ·min^-1^ for 5 min, the pellet on the bottom was resuspended by 1 ml 1640 medium, after that the cell suspension was put in 6 well plate and then incubated 30 min in 37°C in a humidified incubator (5% CO_2_). Pulmonary macrophages were isolated by the adherent method. The numbers of macrophages counting were counted under a microscope at 40× magnification. The measurement of cell infiltration in BALF were measured by Cell-Counting Kit-8 (CCK-8) assays.

### RNA Extraction and Reverse Transcription Polymerase Chain Reaction (RT-PCR)

Total RNA was extracted from lungs using TRIzol reagent (Invitrogen, Carlsbad, CA, USA), and single-strand cDNA was synthesized from total RNA using a Thermoscript RT-PCR synthesis kit (Thermo Fisher Scientific, Waltham, MA USA) according to the manufacturer’s instructions. The glyceraldehyde-3-phosphate dehydrogenase (GAPDH) gene was used as an internal control for normalization. Gene transcription levels were detected with real-time PCR (AB7500, Applied Biosystems, Foster City, CA, USA) using the specific primers, Primer name and sequence were listed as:

GAPDH (Mus musculus):

Forward-5’-CAAGGTCATCCATGACAACTTTG-3’;
Reverse-5’-GTCCACCACCCTGTTGCTGTAG-3’
CHOP (Mus musculus):
Forward-5’-AAGTCTAAGGCACTGAGCGTATC-3’;
Reverse-5’-TTCCAGGAGGTGAAACATAGGTA-3’
TRAIL (Mus musculus):
Forward-5’-GGATGAGGATTTCTGGGACT-3’;
Reverse-5’-CTGCCACTTTCTGAGGTCTT-3’

### Dual Luciferase Gene Reporter Assay

Briefly, 30 μl of the cell lysates were extracted and transferred into 96-well plates (Gronier, 655075). Subsequently, the samples were reacted with the substrates for firefly and Renilla luciferase according to the manufacturer’s instruction (Promega, Madison, WI, USA). Then, a Veritas Microplate Luminometer (Turner Biosystems, Sunnyvale, CA, USA) was used to sequentially detect the activities of firefly (Photinus pyralis) and Renilla (Renilla reniformis) luciferases. The final results were calculated as the fluorescence intensity of firefly luciferase corrected by the Renilla luciferase fluorescence intensity.

### Protein Extraction and Western Blot

Lung tissues were homogenized and lysed using lysis buffer containing protease inhibitor phenylmethylsulfonyl fluoride (PMSF). Protein concentrations were measured using a bicinchoninic acid assay kit. Total sample was separated by 10% sodium dodecyl sulfate-polyacrylamide gel electrophoresis (SDS-PAGE) and then blotted to polyvinylidene fluoride (PVDF) membranes (Merck Millipore, IPVH00010). Membranes were blocked for 2 h at room temperature with 5% bovine serum albumin (BSA). Next, incubate with primary antibodies overnight at 4°C. Horseradish peroxidase-conjugated antibodies against mouse and rabbit were used as secondary antibodies. After extensive washing, blots were developed with an enhanced chemiluminescent plus assay kit (Thermo Scientific), developed on X-ray film, and analyzed by ImageJ software (National Institutes of Health, Bethesda, MD, USA).

### ELISA Assay

The levels of soluble TRAIL, monocyte chemoattractant protein 1 (MCP1), TNF-α, IL-1β, and IL-6 in the broncho alveolar lavage fluid (BALF) were determined using ELISA kits (R&D Systems, Minneapolis, MN, USA) according to the manufacturer’s instructions. ELISA data were obtained in duplicate from at least three independent experiments.

### Cell Apoptosis Detection

The supernatant of THP1 cells treated with PMA and LHQW for 24 h in 6-well plates was transferred to HPA cells (3 × 10^5^/ml). After 24 h, the HPA cells were dissociated by trypsin for apoptosis detection. Caspase3 and AV-PI assays were performed according to the manufacturer’s instructions. Protein concentrations in Caspase3 assay were measured using a bradford protein assay kit according to the manufacturer’s specification.

### Histopathologic Evaluation of the Lung Tissue

Lungs were clipped at the trachea, perfused with 4% paraformaldehyde (PFA), removed, and fixed for 24 h in 4% PFA. Lungs were embedded in paraffin (ASP200S, Leica, Wetzlar, Germany) and cut into 5-μm thick sections. Sections were stained with TUNEL (Takara Biomedicals, Tokyo, Japan) or NKAα1 (clone C464.6, EMD Millipore, Burlington, MA, USA) after antigen retrieval with 10 mM sodium citrate at 95°C for 20 min according to the manufacturer’s instructions. Analyses were performed with ImageJ software.

### Statistical Analysis

Data were analyzed using SPSS 20.0 (IBM, Armonk, NY, USA). Values were presented as mean ± standard deviation. Comparisons among groups were performed using Student’s t tests or one-way analysis of variance. In all cases, *P* < 0.05 was considered statistically significant.

## Results

### Chemical Compounds of LHQW

UPLC was performed to identify the chemical compounds of each herb contained in LHQW. The 12 main active ingredient chemical compositions were as follows: Salidroside (1701.25 μg.g^-1^), Chlorogenic acid (2,492.15 μg.g^-1^), Forsythoside E (1620.78 μg.g^-1^), Cryptochlorogenic acid (1,851.64 μg.g^-1^), Amygdalin (1,455.39 μg.g^-1^), Sweroside (813.18 μg.g^-1^), Hyperin (151.73 μg.g^-1^), Rutin (121.17 μg.g^-1^), Forsythoside A (2,536.34 μg.g^-1^), Phillyrin (1521.45 μg.g^-1^), Rhein (1102.06 μg.g^-1^), Glycyrrhizic acid (1,680.43 μg.g^-1^) (Jia et al., 2015).

### LHQW Significantly Protected Lungs From Inflammatory Damage and Decreased Antibiotic Need in an LPS-Induced ALI Model

**Figure 1:**
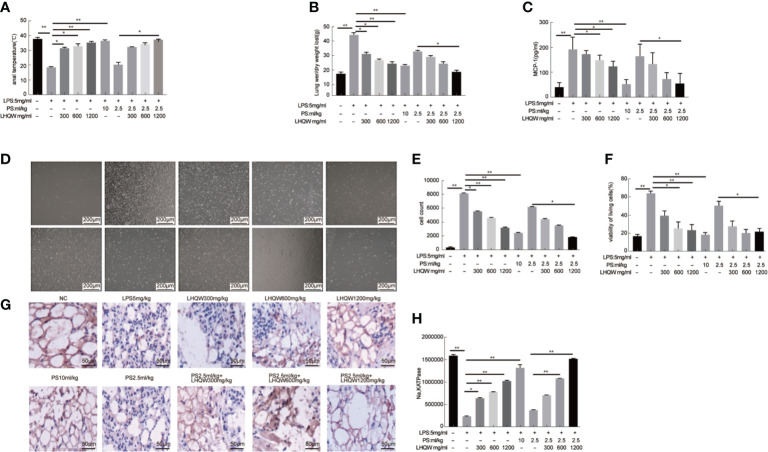
Lian Hua Qing Wen capsule (LHQW) significantly protected lung tissue from inflammatory damage and functioned synergistically with antibiotics in an lipopolysaccharide (LPS)-induced Acute Lung Injury (ALI) model. At 24 h after the last drug treatment, all mice were anesthetized with 4% chloral hydrate (0.4 g.kg^-1^) and placed in the supine position on the surgical board. Control mice were instilled with saline, and others were treated with LPS (5 mg·kg-1). All of the above processes were performed on a ventilator. **(A)** Effects of LHQW on anal temperature. **(B)** Effects of LHQW on lung wet/dry ratio. **(C)** Effect of LHQW on MCP1 secretion. **(D–F)** Effects of LHQW on the measurement of infiltrated cells in BALF were measured with CCK-8 and microscope assays(40×). Lung tissue from ALI model mice were fixed in 4% PFA, then 3-5-μm sections were prepared after paraffin embedding. All sections were subjected to immunohistochemistry. **(G, H)** Effects of LHQW on the degree of edema by measuring Na, K ATPase levels in lung tissue (200×). The data was presented as the mean ± S.E.M.; n = 8 mice per group; *P < 0.05, **P < 0.01 vs. the LPS group.

To evaluate the pharmacodynamics of LHQW, we first measured anal temperature. Compared to the NC group, the anal temperature in the ALI model group decreased to 25.3°C, which was significantly increased after LHQW and PS 10 ml.kg-1 treatment. Notably, there was a synergistic effect of LHQW 1,200 mg.kg-1 combined with PS 2.5 ml.kg-1 compared with PS 2.5 ml.kg-1 alone (**Figure 1A**). Because of the importance of edema for ALI progression and prognosis, we assessed the wet and dry weights for lungs and the ratio. After LPS injection, the ratio was increased nearly 3-fold, but this was significantly attenuated with LHQW and PS 10 ml.kg^-1^ treatment. We also observed a significant increasing in the ratio after LHQW 1,200 mg.kg^-1^ combined with PS 2.5 ml.kg-1 compared with PS 2.5 ml.kg-1 alone (**Figure 1B**). Furthermore, LHQW combined with PS (2.5 or 10 ml.kg-1) treatment were having similar effects. To assess the chemotactic ability of infiltrated cells, MCP1 concentration in BALF was measured by ELISA after LPS treatment. The concentration of MCP1 decreased significantly in mice treated with LHQW. Subsequently, LHQW 1200 mg.kg-1 also had a synergistic effect of PS 2.5 ml.kg-1 compared with PS 2.5 ml.kg-1 alone (**Figure 1C**).

The total infiltrated cells in BALF, which is an indicator of the lung inflammatory response. The numbers were obviously increased by LPS; however, this increase was significantly reduced by LHQW and PS 10 ml.kg-1 treatment. Consistent with our hypothesis, total infiltrated cell numbers were also decreased by at least 2-fold after LHQW 1200 mg.kg-1 combined with PS 2.5 ml.kg-1 compared with PS 2.5 ml.kg-1 alone (**Figures 1D–F**). Alveolar epithelial Na,K ATPases drive the clearance of excess edema fluid, and passive activity of Na,K ATPase in the lung leads to edema due to an imbalance of fluid and salt (Peteranderl et al., 2016). Notably, Na,K ATPase activity was significantly decreased by LPS, but this was reversed by LHQW and PS 10 ml.kg-1. Moreover, LHQW 600, 1,200 mg.kg-1 worked synergistically with PS 2.5 ml.kg-1 to enhance Na,K ATPase activity (**Figures 1G, H**). Collectively, these results demonstrate that LHQW significantly protected lung tissue from inflammatory damages and decreased the required antibiotics dose in an LPS-induced ALI mouse model. LHQW may have a role in decreasing antibiotic abuse and resistance.

### LHQW Inhibited the Expression and Secretion of Inflammatory Molecules and Dampened Inflammatory Responses by Targeting Macrophages *In Vitro* Study

**Figure 2:**
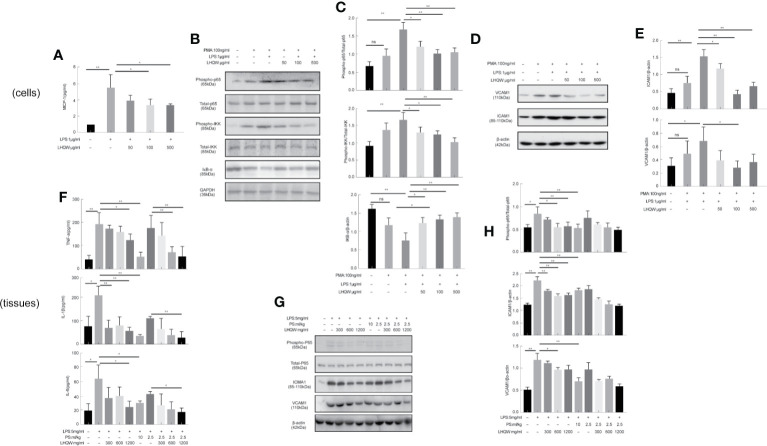
Lian Hua Qing Wen capsule (LHQW) inhibited the expression and secretion of inflammatory molecules in macrophages. Mice were dissected 24 h later, the lungs were lavaged with saline, and BALF was collected. **(A)** Dual luciferase reporter analysis in THP1cells influenced by LHQW. **(B, C)** Detection of NF-κB pathway-related proteins phospho-p65, total-p65, phosphorylation-IKK, total-IKK, and IKB-α in THP1 cells. **(D, E)** Determination of adhesion molecules VCAM1 and ICAM1 by western blot assay. **(F)** LHQW decreased the secretion of TNF-α, IL-1B, and IL-6 measured by ELISA. **(G, H)** The expression of VCAM1, ICAM1, phospho-p65, and total-p65 in alveolar macrophages measured with western blot analysis. The data was presented as the mean ± S.E.M. of three independent experiments. ^#^ *P* < 0.05 vs. the NC group, ^*^ *P* < 0.05, ^**^ *P* < 0.01 vs. the LPS group. LHQW-L: 50 μg/ml; LHQW-M: 100 μg/ml; LHQW-H: 500 μg/ml.

We next measured activity of the transcription factor nuclear factor (NF-κB) (Hamid et al., 2011; Oeckinghaus et al., 2011). We first evaluated the impact of LHQW on NF-κB with dual luciferase reporter gene assays in RAW 264.7 cells. The results clearly showed that LPS-induced macrophages had increased fluorescence intensity in cells transfected with the NF-κB-dependent luciferase reporter construct, subsequently, which was significantly reversed by LHQW (**Figure 2A**). Additionally, the expression of phospho-P65, phospho-IKK, and IKB-α in THP1 cells were detected by western blot. Expression of phospho-P65 and phospho-IKK were significantly upregulated, while IKB-α was obviously downregulated after LPS treatment. These changes were clearly reversed with LHQW treatment (**Figures 2B, C**). Furthermore, we prepared VCAM1 and ICAM1 antibodies (Yang et al., 2015) for western blot analysis in THP1 cells and detected obviously increased expression of both proteins in response to LPS. Both increases were significantly downregulated after LHQW 1,200 mg.kg-1 treatment and VCAM1 was more markedly downregulated than ICAM1 (**Figures 2D, E**).

ELISA assays were performed to assess the concentrations of TNF-α, IL-1β, and IL-6 in BALF. All three pro-inflammatory cytokines were obviously increased in response to LPS (**Figure 2F**). However, we detected marked decreases in TNF-α, IL-1β, and IL-6 concentrations after LHQW 1200 mg.kg-1 and PS 10 ml.kg-1 treatment. TNF-α and IL-1β concentrations were also decreased by LHQW (600 or 1,200 mg.kg-1) combined with PS 2.5 ml.kg-1, and IL-6 concentration was decreased observably by LHQW (1200 mg.kg-1) combined with PS 2.5 ml.kg-1 compared with PS 2.5 ml.kg-1 alone. The expression levels of Phospho-P65, VCAM1, and ICAM1 were consistent with *in vitro* evidence in alveolar macrophages (**Figures 2G, H**). Taken together, these results show that LHQW inhibited the expression and secretion of inflammatory molecules and dampened inflammatory responses by targeting macrophages *in vitro* studies.

### LHQW Alleviated LPS-Induced ERS and Inhibited TRAIL Expression in Macrophages *In Vitro*

**Figure 3:**
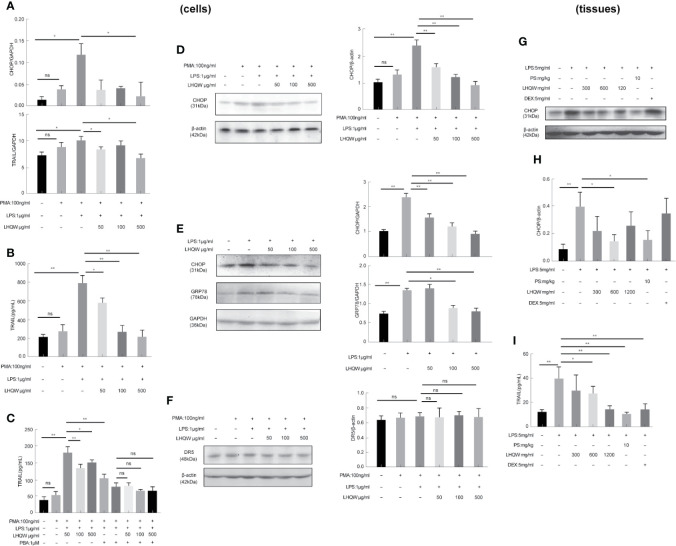
Lian Hua Qing Wen capsule (LHQW) alleviated lipopolysaccharide (LPS)-induced endoplasmic reticulum stress (ERS) and tumor necrosis factor-related apoptosis-inducing ligand (TRAIL) expression in macrophages. Bronchoalveolar lavage fluid (BALF) samples were centrifuged, and pulmonary macrophages were isolated with the adherent method. THP1 cells were first induced by PMA to differentiate into macrophages, and subsequently treated with LHQW and LPS for another 24 h. (Togno-Peirce et al., 2013) In the cells, **(A)** LHQW inhibited the transcription of CHOP and TRAIL in THP1 cells detected by RT-PCR assays. **(B)** LHQW downregulated the expression of TRAIL secretion in the supernatant of THP1 cells detected by ELISA assay. **(C)** Treatment with the ERS inhibitor PBA reduced TRAIL concentration in the supernatant of THP1 cells. **(D, E)** LHQW inhibited the translation of CHOP and GRP78 in THP1 cells and RAW 264.7 cells detected by western blot. **(F)** Effect of LHQW treatment on the expression of DR5, the ligand of TRAIL, in HPA cells treated with the supernatant from THP1 cells. **(G, H)** The translation of CHOP in pulmonary macrophages. **(I)** The secretion of TRAIL in BALF. The data weas presented as the mean ± S.E.M. of three independent experiments. ^#^ *P* < 0.05 vs. the NC group. ^*^ *P* < 0.05, ^**^ *P* < 0.01 vs. the LPS group. LHQW-L: 50 μg/mL; LHQW-M: 100 μg/mL; LHQW-H: 500 μg/mL. GRP78.

To detect the ERS markers CHOP and GRP78, we first prepared CHOP primers (**Table 1**) for reverse-transcription PCR. CHOP expression in LPS-induced THP1 cells was clearly upregulated; however, there was a 5-fold change with LHQW 500 μg/kg treatment. We also measured CHOP and GRP78 in western blot analyses, which obviously upregulated CHOP and GRP78 expression in response to LPS. Both ERS markers were dramatically downregulated by at least 1-fold change after LHQW treatment. Consistent results were obtained in the alveolar macrophages (**Figures 3A1, D–G**). These results demonstrate that LHQW is a candidate for alleviating LPS-induced ERS.

Next, we performed additional reverse-transcription PCR assays and found that and test TRAIL transcription was decreased by LHQW (300, 600 mg.kg-1) treatment compared with LPS-induced THP1 cells (**Figure 3A2**). To measure TRAIL secretion, we performed ELISA assays by using supernatant of THP1 cells and BALF from mice. Both *in vitro* and *in vivo*, TRAIL secretion was clearly upregulated after LPS induction compared with the NC group, but this was dramatically downregulated by LHQW treatment. We hypothesized that TRAIL secretion might be closely related with ERS in macrophages. The ERS inhibitor PBA was used to test this in THP1 cells. There was no significant difference in the response to PBA between the LHQW and LPS groups (**Figures 3B, C**). To identify the specificity of LHQW for an effect on TRAIL, we detected Death Receptor 5 (DR5), the ligand of TRAIL (Liu, et al.) We prepared DR5 antibody for western blot assay in HPA cells that had been treated with supernatant from THP1 cells. DR5 expression was essentially unchanged in response to LPS and LHQW treatment (**Figures 3H, I**). Overall, these results show that LHQW alleviated LPS-induced ERS and TRAIL expression in macrophages *in vitro* and *in vivo*.

### LHQW Significantly Protected Alveolar Epithelial Cells Against TRAIL-Induced Apoptosis in a Macrophage-Epithelial Co-Cultural Model

**Figure 4:**
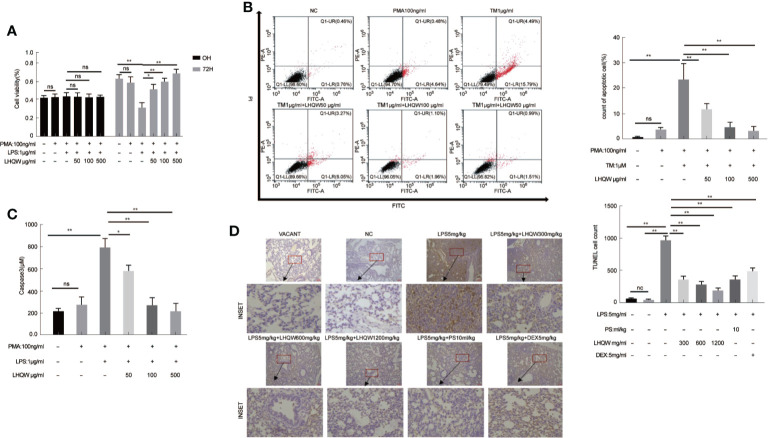
Lian Hua Qing Wen capsule (LHQW) protected alveolar epithelial cells against TRAIL-induced apoptosis. Lung tissue come from ALI model mice were fixed in 4% PFA, then 3-5-μm sections were prepared after paraffin embedding. All sections were subjected to immunohistochemistry. In the macrophage-epithelial co-cultural model, human pulmonary alveolar epithelial cells (HPA cells) in 6-well plates were incubated with supernatant from THP1 cells that were treated with LHQW, tunicamycin (TM), and lipopolysaccharide (LPS). **(A)** The effect of LHQW on increasing the cell counting of HPA cells in the macrophage-epithelial co-cultural model, which was based on MTT assays. **(B, C)** LHQW alleviated apoptosis in the macrophage-epithelial co-cultural model measured by **(B)** AV-PI assay and **(C)** caspase3 activity. **(D)** The number of apoptotic cells (dark brown) in lung tissue measured by TUNEL (200×). THP1 cells in 6-well plates were first treated with PMA for 24 h, followed by LHQW and LPS for another 24 h, then the supernatant was collected. The data was presented as the mean ± S.E.M. of three independent experiments. ^#^ *P* < 0.05 vs. the NC group. ^*^ *P* < 0.05, ^**^ *P* < 0.01 vs. the LPS group. LHQW-L: 50 μg/ml; LHQW-M: 100 μg/ml; LHQW-H: 500 μg/ml; TM: tunicamycin.

Crosstalk between macrophages and epithelial cells eventually leads to disease progression and complications (Peteranderl et al., 2016). Firstly, MTT assays showed that LHQW increased the cell counting of HPA cells in the macrophage-epithelial co-cultural model (**Figure 4A**). AV-PI reagents were added to cells prior to flow cytometry. There were no significant differences in numbers of necrotic epithelial cells (AV^−^PI^+^). TM, which promotes ERS, significantly increased the mean number of HPA cells in early apoptosis (AV^+^PI^−^), which was significantly reduced by LHQW treatment (**Figure 4B**). Next, Capase3 kits were used to determine the mechanism by which LHQW inhibited apoptosis. Mechanistically, caspase3 activity levels were highly consistent with the AV-PI results (**Figure 4C**). To further confirm the antiapoptotic ability of LHQW, TUNEL staining was performed. We observed increased numbers of apoptotic cells in response to LPS 5 mg.kg-1 compared with the NC and VACANT (with no surgery) groups. As expectedly, dramatic reductions in apoptosis were observed after LHQW, PS10 ml.kg-1, and DEX 5 mg.kg-1 treatment (**Figure 4D**). LHQW effectively protected alveolar epithelial cells against TRAIL-induced apoptosis in a macrophage-epithelial co-cultural model.

### LHQW Specifically Inhibited ERS Through JNK Pathway Activation

**Figure 5:**
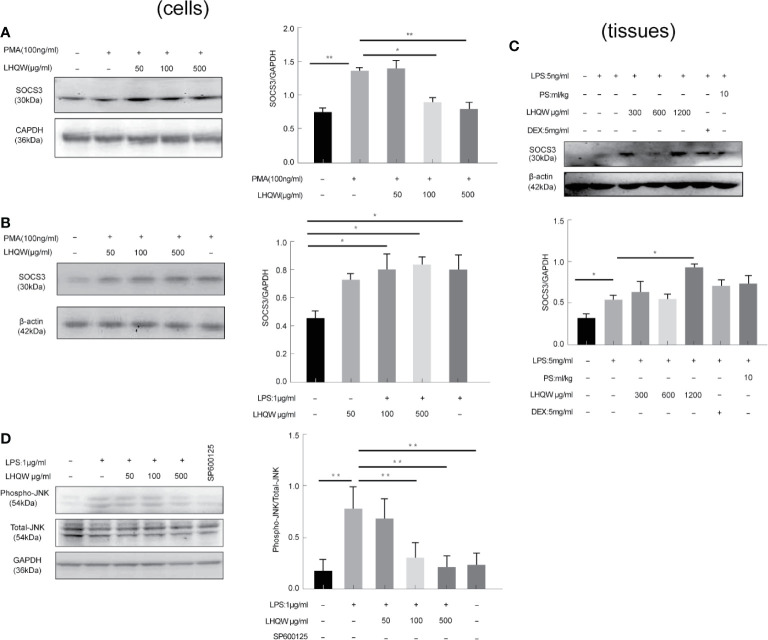
Lian Hua Qing Wen capsule (LHQW) specifically inhibited endoplasmic reticulum stress (ERS) through c-Jun N-terminal kinase (JNK) pathway activation. Bronchoalveolar lavage fluid (BALF) samples were centrifuged, and pulmonary macrophages were isolated with the adherent method. THP1 cells were first induced by PMA to differentiate into macrophages, and subsequently treated with LHQW and LPS for another 24 h. RAW 264.7 cells were also treated with LHQW and LPS for 24 h. (Togno-Peirce et al., 2013). In the cells, **(A, B)** LHQW enhanced SOCS3 expression in **(A)** THP1 cells, **(B)** RAW 264.7 cells by utilizing western blot. **(C)** LHQW decreased phospho-JNK expression by western blot analysis. In the tissues, **(D)** LHQW enhanced the translation of SOCS3 in alveolar macrophages. The data was presented as the mean ± S.E.M. of three independent experiments. ^#^ *P* < 0.05 vs. the NC group. ^*^ *P* < 0.05, ^**^ *P* < 0.01 vs. the LPS group. LHQW-L: 50 μg/ml; LHQW-M: 100 μg/ml; LHQW-H: 500 μg/ml; TM, tunicamycin.

To determine the ability of LHQW to reverse ERS-mediated TRAIL up-regulation, we prepared a SOCS3 antibody for western blot assays in three macrophage lines: THP1 cells, RAW 264.7 cells, and alveolar macrophages. We detected increased expression in response to LHQW treatment in THP1 cells and RAW 264.7 cells (**Figures 5A, B**). Furthermore, consistent with vitro results, SOCS3 expression was upregulated nearly 1-fold in alveolar macrophages from mice treated with LHQW 1,200 mg.kg-1 (**Figure 5C**).

We finally turned our attention to the JNK pathway, which is closely associated with TRAIL transcription and translation. Western blots clearly showed that JNK expression was markedly increased in the LPS group compared to the NC group, but levels were significantly downregulated after LHQW and SP600125 treatment compared with the LPS group (**Figure 5D**). Together, these findings indicate that LHQW attenuated the increase in SOCS3 expression, with the ultimate result of restricting TRAIL production.

## Discussion

Here, we investigated how LHQW affects epithelial cells apoptosis and ERS. By targeting its regulatory effects in the JNK pathway, we revealed that LHQW could decrease SOCS3 expression and as a resultant, restrict TRAIL production in macrophages. Our results provide evidence that LHQW is an effective treatment for ALI and suggests that it could be used as an adjunctive treatment to antibiotics.

Accumulating evidence indicates that ALI is not only the core pathological process of bacterial infection, but also the common pathological process of damage of various respiratory infectious diseases induced by viruses, mycoplasma, fungi and other pathogenic agents. The etiology of respiratory infectious diseases system is caused by the invasion of pathogens or foreign bodies, however, the pathogenic mechanism and damage causes of diseases are more due to the body’s inflammatory imbalance and immune damage(Tabas, 2010; Alessandri et al., 2013; Condamine et al., 2014; Huang et al., 2015). Thus, treatment of respiratory infections diseases have provided insight into pathogen–host interactions, and host-directed therapeutic strategies are becoming feasible adjuncts to standard antimicrobial treatment (Zumla et al., 2016).

Under the background of global outbreak of COVID-19, mortality from the most severe respiratory infections disease remains high. Subsequently, lots of drugs that have once shown promising antiviral efficacy have nearly failed in clinical trials. However, traditional Chinese medicine LHQW capsule can significantly improve the symptoms of COVID-19 suspected cases such as fever, cough, fatigue, shortness of breath, and reduce the proportion of severe cases, providing preliminary clinical evidence for the prevention and treatment of the disease. The facts remind us again that focusing on immunity is of great clinical significance to develop new strategies for the treatment of respiratory infectious diseases, which is a good way to make up for the defects of pathogen inhibition, improve the efficiency of pathogen inhibition and reduce tissue damage. According to the theory of traditional Chinese medicine, we focus on “people” of the disease, highlight the “sick people” and “human diseases” integrated interactions. Newer approaches to improving treatment outcomes would put its insights into pathogen–host interactions, the host’s innate and acquired immune responses, which are leading to identification and development of a wide range of host-directed therapies with different mechanisms of action. Host-directed therapies targeting host immune and inflammatory pathways to enhance immune responses and alleviate immunopathology could benefit treatment outcomes in a range of bacterial, viral, and parasitic diseases(Cheng et al., 2014) (Zumla et al., 2016).

Recent studies showed that the lung’s response to inflammatory conditions was characterized by epithelial injury, pulmonary inflammation, formation of distinctive fibroblasts and excessive extracellular matrix accumulation (Galkina and Ley, 2009). In our study, the tracheal injection of LPS for 24 h can slightly lead to alveolar spaces shrinks and alveolar wall thickening compared to normal group. However, we can hardly observe these symptoms treated with LHQW 1,200 mg.kg-1. We could conclude that LHQW might have potential in resolving pulmonary fibrosis. In addition, some researchers said that pulmonary fibrosis has an indispensable relationship with inflammatory damage and tissue structure destruction (Seimon et al., 2010).

It is well-known that tissue damage is a common feature of many infectious diseases, specifically in ALI (Headland and Norling, 2015). (Freire and Van Dyke, 2013). The interactions between macrophages and epithelial cells, which eventually leads to disease progression, were recently revealed as the therapeutic target in ALI. Subsequently, they can reverse the pathological process of ALI and protect organs from damaging (Hogner et al., 2013; Peteranderl et al., 2016). Under the inflammatory conditions, TRAIL facilitated the inflammatory reaction that promotes unspecific tissue injury and disease severity. TRAIL is closely related to the ERS response and might activate DRs on target cell membranes or alter intracellular pathways, and the pathogen itself might exploit TRAIL-induced pathways for its own survival and replication (Tiwary et al., 2010; Liu et al., 2016). Therefore, TRAIL or its downstream signaling events might reflect ALI severity. Importantly, the regulation of TRAIL expression in the setting of ERS was accompanied by a significant decrease in SOCS3 (Kim et al., 2013; Chen et al., 2015) (Huang et al., 2015). In summary, our studies suggested that LHQW can enhance SOCS3 expression to reverse ERS-induced aberrant expression of TRAIL through JNK pathway, which is closely related with ERS-induced transcription and translation of TRAIL. Based on the analysis above, we concluded that LHQW have potential in alleviating tissue damage induced by TRAIL and it just provide new insight to infectious disease.

In conclusion, the present study demonstrated that LHQW effectively ameliorated inflammatory damage, blocking ALI progression. Furthermore, we need a systematic analysis to determine how LHQW regulate the immune injury and pharmacologically protect tissues from damaging.

## Data Availability Statement

The datasets generated for this study are available on request to the corresponding author.

## Ethics Statement

The animal study was reviewed and approved by Laboratory Animal Ethics Committee of the Institute of basic theory of traditional Chinese medicine, of the China Academy of Chinese Medical Sciences (license number SCXK 2016-0021).

## Author Contributions

QR and QL participated in the manuscript writing and experimental design and performed the experiments. LS, JY, TL, LL, and ZZ participated in the experiments. QY, YL, YC, XW, YW, and WC participated in the manuscript writing, manuscript editing, and data analysis. XZ participated in the manuscript writing, experimental design, and data analysis. All authors contributed to the article and approved the submitted version.

## Funding

This work was supported by grants from the National High Technology Research and Development Program of China (2017ZX09101002-002-002); the Institute of Traditional Chinese Medicine internal project—the research on reevaluating traditional Chinese medicine based on antibiotic substitution (No. Z2017019-03); “the Belt and Road” cooperation project of China academy of traditional Chinese medicine (No. GH201914); The General Programs of the National Natural Science Foundation of China (8157141621); Special training program for outstanding young scientific and technological talents of China academy of Chinese Medical Science (ZZ13-YQ-044); Independent topic selection project China Academy of Chinese Medical Science (ZXKT17010); China Academy of Traditional Chinese Medicine internal project—Construction of China-European research center for traditional Chinese medicine and natural products (No. GH2017-01).

## Conflict of Interest

The authors declare that the research was conducted in the absence of any commercial or financial relationships that could be construed as a potential conflict of interest.

## Abbreviations

ALI, acute lung injury; AP-1, activator protein 1; AV-PI, annexin V-propidium iodide; BALF, bronchoalveolar lavage fluid; Caspase3, cysteine-requiring aspartate protease 3; CHOP, DNA damage inducible transcript 3; DEX, dexamethasone; ELISA, enzyme-linked immunosorbent assay; ERS, endoplasmic reticulum stress; HPA cell, human pulmonary alveolar epithelial cells; ICAM1, intercellular adhesion molecule 1; IFN, interferon; IKB-α, inhibitor of nuclear factor kappa-B kinase-α; IKK, inhibitor of nuclear factor kappa-B kinase; IL-1β, interleukin 1 beta; IL-6, interleukin 6; JNK, c-Jun N-terminal kinase; LHQW, Lian Hua Qing Wen capsule; LPS, lipopolysaccharide; MTT, methyl thiazolyl tetrazolium; NC, negative control; P-65, RELA proto-oncogene; PMA, phorbol-12-myristate-13-acetate; PS, penicillin-streptomycin; SOCS3, suppressor of cytokine signaling 3; THP1 cell, human acute monocytic leukemia cell line; TM, tunicamycin; TNF-α, tumor necrosis factor alpha; TRAIL, tumor necrosis factor-related apoptosis-inducing ligand; TUNEL, terminal-deoxynucleotidyl transferase-mediated nick end labeling; VCAM1, vascular cell adhesion molecule 1.
