# Supplementary material for: Lian Hua Qing Wen Capsules, a Potent Epithelial Protector in Acute Lung Injury Model, Block Proapoptotic Communication Between Macrophages, and Alveolar Epithelial Cells
Source: Front Pharmacol. 2020 Sep 23;11:522729. doi: 10.3389/fphar.2020.522729 (PMC7538620; doi:10.3389/fphar.2020.522729)
Supplement: Supplementary file 1 [file DataSheet_1.docx]

**concentration/ratio of the plants present in the preparation:**

LHQW is composed of 11 herbs including Forsythia suspensa (Thunb.) Vahl (Lianqiao, 255g); Lonicera japonica Thunb. (Jinyinhua, 255g); Honey-fried Ephedra sinica Stapf (Mahuang, 85g); Prunus armeniaca L. (Kuxingren, 85g); Isatis tinctoria L. (Banlangen, 255g); Dryopteris crassirhizoma Nakai (Mianmaguanzhong, 255g); Houttuynia cordata Thunb. (Yuxingcao, 255g); Pogostemon cablin (Blanco) Benth. (Guanghuoxiang, 85g); Rheum palmatum L. (Dahuang, 51g), Rhodiola crenulata (Hook.f. & Thomson) H.Ohba (Hongjingtian, 85g); and Glycyrrhiza glabra L. (Gancao, 85g); along with menthol (Bohenao, 7.5g)and a traditional Chinese mineral medicine, Gypsum Fibrosum (Shigao, 255g).
